# Sex chromosome loss after allogeneic hematopoietic stem cell transplant in patients with hematologic neoplasms: a diagnostic dilemma for clinical cytogeneticists

**DOI:** 10.1186/s13039-016-0275-3

**Published:** 2016-08-08

**Authors:** Zhenya Tang, L. Jeffrey Medeiros, C. Cameron Yin, Wei Wang, Xinyan Lu, Ken H. Young, Joseph D. Khoury, Guilin Tang

**Affiliations:** Department of Hematopathology, The University of Texas MD Anderson Cancer Center, 1515 Holcombe Boulevard, Houston, TX 77030-4009 USA

**Keywords:** Sex chromosome loss (SCL), –X, –Y, Allogeneic hematopoietic stem cell transplant (alloHSCT), Chimerism testing, Cytogenetic (CG) analyses

## Abstract

**Background:**

Sex chromosome loss (SCL), including loss of an X chromosome (-X) in females and loss of the Y chromosome (-Y) in males, resulting in a karyotype of 45,X, rarely occurs in patients post an allogeneic hematopoietic stem cell transplant (alloHSCT). However, origin of this abnormal clone and its clinical significance remains unknown.

**Results:**

We present 12 cases with SCL who underwent alloHSCT; 9 patients (4 men and 5 women with a median age of 56 years) developed isolated SCL after alloHSCT (Group I), and 3 patients (all women with a median age of 58 years) had a SCL before undergoing alloHSCT after which SCL disappeared (Group II). The primary neoplasms included chronic lymphocytic leukemia (*n* = 5), acute myeloid leukemia (*n* = 5), chronic myelogenous leukemia with nodal marginal zone lymphoma (*n* = 1) and Hodgkin lymphoma (*n* = 1). According to the donor/recipient relationship, their alloHSCT can be divided into sex-matched, HLA-matched, unrelated donors (*n* = 2); sex-mismatched, HLA-matched, unrelated donors (*n* = 4); sex-mismatched, HLA-matched, related donors (2 HLA-identical and 2 HLA-haploidentical cases) and sex-matched, HLA-matched, related donors (2 HLA-haploidentical cases). In Group I, isolated SCL was first detected with a median interval of 3 months (range 1 to 42 months) after the alloHSCT. By the end of clinical follow-up in patients in Group I, 7 patients expired with a median overall survival of 45 months (range 3 to 108 months) after alloHSCT and 33 months (range 0 to 66 months) after SCL detection. In Group II, 1 patient expired with a survival time of 54 months after the alloHSCT. Detection of SCL after alloHSCT can be transient, intermittent or persistent.

**Conclusions:**

Interpretation of SCL is challenging in the context of alloHSCT. Chimerism testing is useful in determining the origin of SCL. In the case of SCL with donor/recipient chimerism, deduction of the SCL origin by all means and use of “–?X” or “–?Y” in the ISCN nomenclature are recommended. Clinical follow-up with closely monitoring the SCL by both cytogenetic and molecular analyses is needed.

## Background

Sex chromosome loss (SCL), defined as loss of an X chromosome in a female (minus X or –X) or loss of the Y chromosome (minus Y or -Y) in a male, is commonly observed in healthy individuals with advancing age. It is considered to be an age-related nondisjunction phenomenon. However, in patients with various hematologic malignancies, detection of –X or –Y has been interpreted, at least in some cases, as evidence of an abnormal clonal karyotype, although the clinical impact of SCL as a biomarker for a hematologic malignancy is elusive [1–11].

Human leukocyte antigen (HLA) matched hematopoietic stem cell transplant (HSCT) is considered a highly effective treatment or even a permanent cure for several hematologic malignancies. This type of therapy is widely applied in the clinical practice, e.g., there have been over 1 million HSCTs performed between 2006 and 2014 worldwide [12–14], and 19,220 HSCTs (10,872 autologous (autoHSCTs) and 8348 allogeneic (alloHSCTs)) in the United States in 2013 alone [15]. Due to a possible co-existence of cells originated from both donor and recipient, interpreting the finding of a SCL after an alloHSCT can be very challenging, depending on multiple factors, such as age of donor and recipient, type of alloHSCT (sex-matched vs. sex-mismatched; related vs. unrelated donor; identical vs. haploidentical), level of chimerism, technical limits of methods used for detection of SCL and other factors.

Here we report 9 patients who developed isolated SCL after an alloHSCT, and their donor types included sex-matched, unrelated; sex-mismatched, unrelated; sex-mismatched, related HLA-haploidentical and sex-mismatched, related HLA-identical. We also report 3 additional patients who had SCL prior to alloHSCT after which SCL disappeared which we consider as a control group. We focus on the challenges of interpreting SCL in these complicated clinical scenarios, along with our recommendations for reporting and interpreting the SCL findings.

## Methods

### Patients

We searched the Clinical Cytogenetics database in the Department of Hematopathology at The University of Texas MD Anderson Cancer Center for –X or –Y as the sole chromosomal abnormality in patients with a history of allogeneic SCT from November, 2000 through December, 2015. Patients with any other chromosomal abnormalities in addition to –X or -Y were excluded from this study. Detailed clinical and laboratory information were collected and reviewed following institutional guidelines with informed consent in accord with the Declaration of Helsinki.

### Conventional chromosomal analysis

Conventional chromosomal analysis (karyotyping) was performed on G-banded metaphase cells prepared from unstimulated 24-h (24 h) and 48-h (48 h) bone marrow cultures as reported previously [11]. Routinely, 20 metaphases were analyzed per case, but less or more than 20 metaphases were analyzed in some cases, due to either an inadequate metaphases available for a complete analysis or a request by a Cytogeneticist for more intensive analysis. Clonal SCL was defined as –X or –Y detected in 3 or more cells of the same analysis, and only patients with clonal SCL were included in this study. All results were reported according to the International System for Human Cytogenetic Nomenclature 2013 (ISCN2013) guidelines [16].

### Fluorescence in situ hybridization (FISH) analysis

The Vysis XY dual color probe set, SpectrumOrange Probe targeting Xp11.1-q11.1 alpha satellite DNA, DXZ1 and SpectrumGreen Probe targeting Yq12 satellite III DNA, DYZ1 (Abbott Molecular, Des Plaines, IL) was used for FISH analysis following established procedures in the Clinical Cytogenetics Laboratory [11]. A total of 400 interphases was counted in each case by two readers.

### PCR-based microsatellite polymorphism (chimerism) analysis

PCR-based microsatellite polymorphism analysis was performed on specimens collected from donors and recipients. Briefly, genomic DNA was extracted from bone marrow aspirate specimens and subjected to one multiplex-PCR amplification of 8 informative short tandem repeat (STR) markers (D6S264, D3S1282, D18S62, D3S1300, DM1, AR, D11S987 and D9S171). The fluorescence-labeled PCR products were subsequently fractionated on an ABI 3100 Genetic Analyzer and analyzed using GeneScan 2.1 software (Thermo Fisher Scientific, Waltham, MA). Patients were followed after alloHSCT at various intervals according to different protocols at MD Anderson over the years. The post-transplant STR information of recipient was compared with the pre-transplant STR information of both donor and recipient to evaluate for the level of chimerism.

## Results

### Patients and allogeneic hematopoietic stem cell transplants (alloHSCTs)

The study is composed of two groups of cases. Patients in Group I (cases 1 to 9) had acquired isolated clonal SCL after alloHSCT. Patients in Group II (cases 10 to 12) had isolated SCL before alloHSCT (Table 1). The 9 patients in Group I were 4 men and 5 women with a median age of 56 years (range 26-69 years) when isolated SCL was detected after alloHSCT. The primary diseases of these patients included chronic lymphocytic leukemia (CLL) in 4 cases; acute myeloid leukemia (AML) in 3 cases; chronic myelogenous leukemia (CML) with nodal marginal zone lymphoma in 1 case; and Hodgkin lymphoma in 1 case. In this group, 2 patients received alloHSCT from sex-matched, HLA-matched, unrelated donors (cases 1 and 8) and 7 patients (cases 2 to 7 and 9) from sex-mismatched donors; 4 received alloHSCT from unrelated donors (cases 2 and 5 to 7), 3 from related donors (2 from HLA-identical sister, cases 3 and 4; and 1 from HLA-haploidentical father, case 9). Four patients (cases 1, 2, 5 and 6) received alloHSCT from anonymous donors through the Matched Unrelated Donor Transplant (MUD) program and ages of these donors are not available. In Group II, 3 female patients had a median age of 58 years (range 19-66 years) when isolated SCL was initially detected prior to alloHSCT. Their SCL disappeared after the alloHSCT. In this group, all 3 patients received alloHSCT from HLA-haploidentical related donors (2 sex-matched, 1 sex-mismatched). In this study, all patients received a BM alloHSCT except one (case 7) who received 2 separate PB alloHSCTs from the same donor. Her SCL had occurred after the second PB alloHSCT. The clinical features and donor information for each patient listed in Table 1.

**Table 1:** Clinical features of recipient/donor and laboratory results

	Recipient Information				Donor Information			First SCL detection			Follow-up and outcomes					
	Case	Sex	Age^a^	Primary disease	Sex	Age^b^	Relationship with donor	Interval^c^	CG analysis	Chimerism test	Interval^d^	Last CG analysis	Last Chimerism test	GVHD	Relapse	Outcome
Group I	1	F	52	CML + NMZL	F	n/a	Unrelated	1	//45,X,-X [3]/46,XX[17]	Donor 100 %	12	//45,X,-X [4]/46,XX[16]	Donor 100 %	Yes	Yes	Dead
	2	M	64	CLL	F	n/a	Unrelated	1	45,X,-?Y[3]/46,XY[5]//46,XX[3]	Recipient 72 %; donor 28 %	41	nuc ish(DXZ1,DYZ1)x1[381/400]//DXZ1x2)[19/400]	Recipient 67 %; donor 33 %	No	Yes	Dead
	3	M	69	CLL	F	53	HLA-identical, related	19	45,X,-?Y[8]/46,XY[22]//	Recipient 82 %; donor 18 %	33	46,XY,del(5)(q13q33)[10]/46,XY[8]//.nuc ish(DXZ1x1,?DZY1x0)[15/400]/(DXZ1,DYZ1)x1[310/400]//(DXZ1x2)[75/400]	NA	No	Yes	Dead
	4	M	56	CLL	F	58	HLA-identical, related	2	45,X,-?Y[3]/46,XY[13]//46,XX[4]	Recipient 38 %; donor 62 %	58	45,X,-Y[5]/45,X,-Y,del(13)(q12q14)[2]/46,XY,del(13)(q12q14)[2]/46,XY[11]//	Recipient 100 %	No	Yes	Dead
	5	F	69	CLL	M	n/a	Unrelated	3	//45,X,-Y[3]/46,XY[17]	Donor 100 %	0	NA	NA	Yes	No	Dead
	6	M	44	AML	F	n/a	Unrelated	2.5	//45,X,-X[3]/46,XX[17].//nuc ish(DXZ1x1)[27/400]/(DXZ1x3)[8/400]/(DXZ1x2)[365/400]	Donor 100 %	5	//nuc ish(DXZ1x1)[24]/(DXZ1x2)[376/400]	Donor 100 %	Yes	No	Dead
	7	F	67	AML	M	72	Unrelated	42	//45,X,?-Y[3]/46,XY[17].//nuc ish(DXZ1x1,DZY1x0)[30/400]/(DXZ1,DZY1)x1[370/400]	Donor 100 %	66	46,XX,del(7)(q31q34),inv(12)(q22q24)[18]//46,XY[3]	NA	Yes	Yes	Dead
	8	F	38	Hodgkin lymphoma	F	23	Unrelated	13	//45,X,-X[4]/46,XX[16]	Donor 100 %	11	//45,X,-X[1]/46,XX[19]	Donor 100 %	No	No	ACR
	9	F	26	AML	M	50	HLA-haploidentical, related	24	//45,X,-Y[5]/46,XY[15]	Donor 100 %	38	//45,X,-Y[10]/46,XY[10]	Donor 100 %	No	No	ACR
Group II	10	F	19	AML	M	42	HLA-haploidentical, related	-2^e^	45,X,-X,inv(9)(p12q13)[7]/46,XX,inv(9)(p12q13)[13].nuc ish(DXZ1x1)[91/400]	Donor 100 %^f^	12^g^	//46,XY[20]	Donor 100 %	Yes	No	ACR
	11	F	66	CLL	F	64	HLA-haploidentical, related	-1^e^	45,X,-X,inv(9)(p12q13)[6]/46,XX,inv(9)(p12q13)[14]	Donor 100 %^f^	54^g^	Complex karyotype with inv(9)(p12q13)//	Recipient 38 %; donor 62 %	Yes	Yes	Dead
	12	F	58	AML	F	51	HLA-haploidentical, related	-12^e^	46,XX,t(8;21)(q22;q22)[14]/45,idem,-X[6]	Donor 100 %^f^	13^g^	//46,XX[20]	Donor 100 %	No	No	ACR

^a^ Recipient’s age at the first detection of SCL after alloHSCT

^b^ Donor’s age at BM/PB HSC donation

^c^ Interval between alloHSCT and the first detection of SCL (months)

^d^ Interval between the first detection of SCL and the last visit or death (months)

^e^ Interval between first detection of SCL and alloHSCT (months)

^f^ Interval between alloHSCT and the first chimerism testing (months)

^g^ Interval between alloHSCT and the last visit or death (months)

*NMZL* nodal marginal zone lymphoma

### Cytogenetic analysis

In Group I, all patients had a normal diploid karyotype through preparative therapy (e.g., chemotherapy of various regimens) prior to alloHSCT. An isolated SCL was first detected with a mean interval of 12 months (range, 1 to 42 months) after the alloHSCT. The clone size of SCL was 15 to 25 % or 3 to 5 metaphases per 20-metaphase analysis in all 9 patients. One patient (case 5) died within one month after the detection of SCL, whereas the other patients have been followed-up for a mean interval of 33 months (range 5 to 66 months). During follow-up all 8 patients had at least 2 more conventional cytogenetic analyses, and/or FISH analysis. The SCL was detected consistently in 3 patients (cases 1, 3 and 4), intermittent in 3 patients (cases 6, 8 and 9) and was not detected in subsequent specimens in 2 patients (cases 2 and 7) (data not included). The most recent conventional cytogenetic analysis in case 8 detected a –X in one metaphase; although not a clonal abnormality, it is still included in the nomenclature for the purpose of documentation as well as reference for future analyses.

In Group II, initial SCL detection was 2, 1 and 12 months prior to alloHSCT in cases 10, 11 and 12, respectively. In addition to SCL, cases 10 and 11 had both inv(9)(p12q13) which is considered as chromosomal polymorphism found in the general population without known clinical significance, but can be used as marker to distinguish recipient and donor cells. Case 12 had a t(8;21)(q22;q22). After alloHSCT, the SCL disappeared in all 3 cases during a follow-up interval of 12 to 54 months. One patient (case 11) developed disease relapse and died whereas two patients (cases 10 and 12) remained as a complete remission.

### Microsatellite polymorphism (chimerism) analysis

In Group I, the bone marrow aspirate specimen showed 100 % donor cells at time of first detection of SCL in 6 patients (cases 1 and 5-9). Therefore, their SCL was considered to originate from donor cells. This status of 100 % donor cells remained unchanged in 4 patients (cases 1, 6, 8 and 9) during a follow-up of 12, 5, 11 and 38 months, respectively, but converted into a chimerism with a mixture of donor and recipient cells at the end of follow-up of 66 months in case 7 (based on the latest cytogenetic analysis). One patient (case 5) died shortly after the detection of SCL. Bone marrow aspirate specimens collected from 3 patients (cases 2, 3, and 4) showed chimerism with a mixture of donor and recipient cells when SCL was initially detected. During a follow-up of 33 to 58 months, the chimerism persisted in two patients (cases 2 and 3), but converted into 100 % recipient cells in case 4 (based on both conventional cytogenetic analysis and chimerism tests). In group II, a status of 100 % donor cells after alloHSCT remained unchanged in two patients (cases 10 and 12) during a follow-up of 12 and 13 months; 100 % donor cells was reached, but shortly thereafter converted to chimerism in one patient (case 11).

### Follow-up and outcome

At last clinical follow-up, 7 patients had died (cases 1 to 7) and 2 patients remain alive in complete remission (cases 8 and 9) in Group I. For the 7 deceased patients in this group, their mean survival time were 32 months (range 3 to 108 months) after the alloHSCT and 24 months (range 0 to 66 months) after detection of SCL. Four patients (cases 1, 5-7) had developed graft-versus-host disease (GVHD), 5 patients (cases 1 to 4-7) had a relapse of primary disease, and one patient (case 5) had persistent primary disease (CLL) after alloHSCT (Table 1). In Group II, 1 patient died (case 11) and 2 patients were alive with complete remission (cases 10 and 12). Two patients had a GVHD (cases 10 and 11) and 1 had a relapse of primary disease (case 11).

## Discussion

When sex chromosome loss (SCL) after alloHSCT is encountered, one of the biggest challenges is to determine the origin of the SCL. Is the SCL originally from the donor or the recipient cells? Is the SCL actually a -X or a -Y in a sex-mismatched alloHSCT case?

Determination of the origin of cells with chromosomal aberration(s) is very important in a predicted negative event, such as disease relapse, graft rejection and GVHD, or donor cell hematologic malignancies [17, 18] and is helpful for determining appropriate intervention or therapy [19]. However, for isolated SCL after alloHSCT, neither karyotyping nor FISH analyses are useful to determine the origin of SCL. A positive SCL result detected by one of these two methods is usually described as “–X or –Y”. In contrast, microsatellite polymorphism analysis, by detecting short tandem repeat (STR) markers in the engraftment (chimerism test), is very useful to address the question of origin. In Group I of this study, the origin of SCL was deduced by the chimerism testing results indicating either 100 % donor cell origin or 100 % recipient origin in 6 patients (cases 1, 5-9) at the time SCL was detected initially and in 5 patients (cases 1, 4, 6, 8 and 9) at the last CG analysis and chimerism test. The “-X” or “-Y” in the ISCN description in Table 1 was concluded from a combination of the CG analysis and chimerism test results. It must be noted that both sensitivity and specificity of chimerism testing can be impacted by multiple factors, for example, relationship of donor and recipient (e.g., monozygotic twins), number and selection of STR markers, and specimen quality as well as other factors [20, 21].

For an ideally successful alloHSCT, the recipient’s hematopoietic and lymphoid cells in bone marrow are replaced by cells derived from the donor HSCs. However, a status of recipient-donor chimerism (henceforth referred to as chimerism), in other words co-existence of recipient and donor cells, can be possible in certain cases. Patients without a pre-alloHSCT ablative preparation and patients having a post-alloHSCT relapse are especially susceptible to developing chimerism in their bone marrow [19]. In this study, 3 patients (cases 2-4) exhibited chimerism at the time SCL was initially detected and 4 patients (cases 2, 3, 7 and 11) at the last post-alloHSCT follow-up tests (CG analysis and/or chimerism test). The origin of SCL in these cases (except case 11) remains unknown. For these cases, writing up an appropriate nomenclature can be challenging for a Cytogeneticist. The previous descriptions of “–X or –Y” is not able to set the clonal SCL in an appropriate spot of the ISCN description where a “//” is used to separate recipient karyotype(s) from donor karyotype(s). Therefore, we recommend the following rules: 1. use “–?X” or “–?Y” instead of “–X or –Y” to describe the SCL. In this way, the SCL can be classified as part of recipient cells (e.g., cases 2-4) or donor cells (e.g., case 7) in sex-mismatched alloHSCT cases; 2. Deduce the most likely origin of SCL in cases with a chimerism by correlating previous SCL results, other additional chromosomal aberration(s) and chimerism test results. For example, SCL plus a polymorphism (such as inv(9) in cases 10 and 11) or a recurrent chromosome aberration which had presented in the recipient previously, usually indicates that the origin of SCL is most likely recipient cells. In this study, the origin of SCL was sustained in all cases with a status of 100 % donor cells (cases 1, 6 and 9) and a converted status of 100 % recipient cells (case 4) during follow-up. A conversion of SCL origin from recipient to donor cells or vice versa was not been observed in this study. Therefore, a deduction of SCL origin with a “–?X” or a “–?Y” may help for better monitoring cases with chimerism.

The causes of SCL in patients with hematologic malignancies can be multifactorial. As we reported recently [11], an age-related “physical event”; selective advantage of SCL clone; hematologic malignancies themselves and certain interventions, such as chemotherapy, all can cause SCL. In this study, several patients and donors were ≥ 60 years of age when the alloHSCT was performed and therefore an age-related nondisjunction phenomenon may play a role. In addition, all patients in this study were treated with and subsequently weaned off immunosuppressive agents to prevent GVHD; 6 patients had GVHD of various severity. As hypothesized by other research groups [22, 23], immunosuppressive intervention and GVHD could cause SCL in these patients as well.

Five patients from Group I and 1 patient from Group II had a relapse of disease; 7 patients from Group I and 1 patient from Group II died during follow-up. Due to the heterogeneity of disease and clinical course in each patient as well as the limited number of cases available for stratification and further analyses in this study, it is difficult to correlate SCL with outcomes. Therefore, interpreting the clinical significance of SCL after alloHSCT is another challenge for a Cytogeneticist. It has been reported previously that a clonal -Y resulting in a loss of minor histocompatibility antigens on the Y chromosome (HY) is associated with loss of graft-versus-leukemia (GVL) effect and hematologic relapse in male patients with myeloid or lymphoid leukemia after a sex-mismatched alloHSCT [23]; even a mosaic loss of chromosome Y in peripheral blood can be associated with shorter survival and higher risk of cancer as was shown in a huge population-based study [24]. Loss of X chromosome (-X) may be disease-associated in patients with myeloid malignancies [11]. Therefore, we recommend that SCL after alloHSCT be considered as a clonal abnormality with potential clinical significance. Instead of immediate intervention, close monitoring of the SCL by both cytogenetic and molecular analyses seems warranted.

## Conclusions

In summary, determining the origin of an acquired isolated SCL after alloHSCT can be challenging to Cytogeneticists. The clinical impact of isolated SCL can be difficult; for some patients, careful follow up seems indicated. Lastly, integrating SCL into an appropriate ISCN nomenclature is also challenging and we suggest a minor change in terminology to accommodate this issue.

## Abbreviations

alloHSCT, allogeneic hematopoietic stem cell transplant; AML, acute myeloid leukemia; autoHSCT, autologous hematopoietic stem cell transplant; CG, cytogenetic; CLL, chronic lymphocytic leukemia; CML, chronic myelogenous leukemia; GVHD, graft-versus-host disease; HLA, the human leukocyte antigens; HSCT, hematopoietic stem cell transplant; MUD, matched unrelated transplant; NMZL, nodal marginal zone lymphoma; SLC, sex chromosome loss; STR, short tandem repeat
